# Frequently assessed and used prognostic factors for outcome after macular hole surgery: which is better?

**DOI:** 10.1186/s12886-021-02164-2

**Published:** 2021-11-18

**Authors:** M. Roth, N. Schön, L. Jürgens, D. Engineer, K. Kirchhoff, R. Guthoff, J. Schmidt

**Affiliations:** 1 Department of Ophthalmology, Heinrich-Heine University Düsseldorf, Universitätsaugenklinik Düsseldorf, Moorenstr. 5, 40225 Düsseldorf, Germany; 2 Augenklinik Tausendfensterhaus, Duisburg, Germany; 3 Department of Ophthalmology, University Clinic Marburg, Marburg, Germany

**Keywords:** Macular hole, Prognostic parameters, Vitreoretinal surgery, Optical coherence tomography, Vitrectomy

## Abstract

**Background:**

The aim of this retrospective study was to evaluate commonly used clinical and OCT-morphological parameters, including perifoveal pseudocysts, as prognostic factors for postoperative outcome after macular hole surgery in a retinal referral clinic in North Rhine-Westphalia, Germany.

**Methods and material:**

This was a retrospective analysis of all patients who underwent surgery because of idiopathic MH between 2011 and 2017 in Augenklinik Tausendfensterhaus, Duisburg, Germany. Statistical evaluation of clinical and OCT-based parameters, including the areas of intraretinal pseudocysts, was conducted. The main statistical outcomes were surgical success and visual acuity. Only parameters with a highly significant correlation to the outcome parameters (postoperative visual acuity (VA); surgical success) in univariate analysis were entered in linear and logistic regression analyses.

**Results:**

A total of 189 eyes of 178 patients (71.4% female; mean age 67.5 ± 8.2 a) who underwent surgery because of MH were included. The overall closure rate was 86.8%. The mean best corrected VA increased from 0.7 ± 0.3 logMAR before surgery to 0.5 ± 0.3 logMAR (*p* < 0.0001). While several clinical and OCT-based parameters as well as calculated indices showed a significant correlation with the outcome measures, the regression analysis showed that the minimum linear diameter was the only parameter that both predicted surgical success (*p* = 0.015) and was correlated with postoperative VA (*p* < 0.001).

**Conclusion:**

The minimum linear diameter serves as an easily assessed prognostic factor with the best predictive properties. This result is of great importance for clinical practice, as it simplifies the postsurgical prognosis.

## Introduction

A full-thickness macular hole (MH) causes a reduction in visual acuity, central visual field scotoma and possibly metamorphopsias. Spontaneous closure is rare (4 to 11.5% [1]); thus, MH usually needs to be treated surgically by pars plana vitrectomy with fluid-gas exchange and epiretinal and internal limiting membrane peeling. In a recent database study by Steel et al. with more than 1200 included operations, the success rate (corresponding to anatomical closure of the MH) of surgical therapy reached almost 96% [2]. In addition to common clinical parameters (e.g., age, duration of symptoms and preoperative visual acuity (VA)), several parameters measured by optical coherence tomography (OCT), such as minimum linear diameter, basal diameter, MH height, and indices based on those OCT measurements (macular hole index (MHI), diameter hole index (DHI) and tractional hole index (THI)), have been proposed as possible prognostic factors for surgical outcome and postoperative visual acuity [2–8] Additionally, the quantification of perifoveal pseudocysts might serve as a prognostic factor, but the relevant literature regarding this parameter is very limited thus far [9, 10]. Opinions on which of those parameters might be the best prognostic factor are controversial. Thus, the aim of this retrospective study was to evaluate these commonly used clinical and OCT-morphological parameters as well as perifoveal pseudocysts as prognostic factors for postoperative outcome (anatomical closure of MH, postoperative VA) in a relatively large cohort of patients with MH treated by surgeons in a busy retinal referral clinic in North Rhine-Westphalia, Germany.

## Methods and material

Before initiation of this retrospective study, approval was obtained from the North Rhine Medical Association (Ärztekammer Nordrhein). The study adhered to the tenets of the Helsinki Declaration. In the inhouse registries of the eye clinic, all patients who had been treated because of MH from the 1st of January 2011 to the 31st of December 2017 were identified.

### Inclusion/exclusion criteria

Only patients with an idiopathic full thickness macular hole regardless of axial length were included. Patients with lamellar macular holes were excluded. Furthermore, posttraumatic macular holes and patients with any other retinal disorder (e.g., AMD or diabetic retinopathy) or with previous retinal surgeries (e.g., after retinal detachment) or intravitreal injections were excluded.

### Parameters

**Fig. 1:**
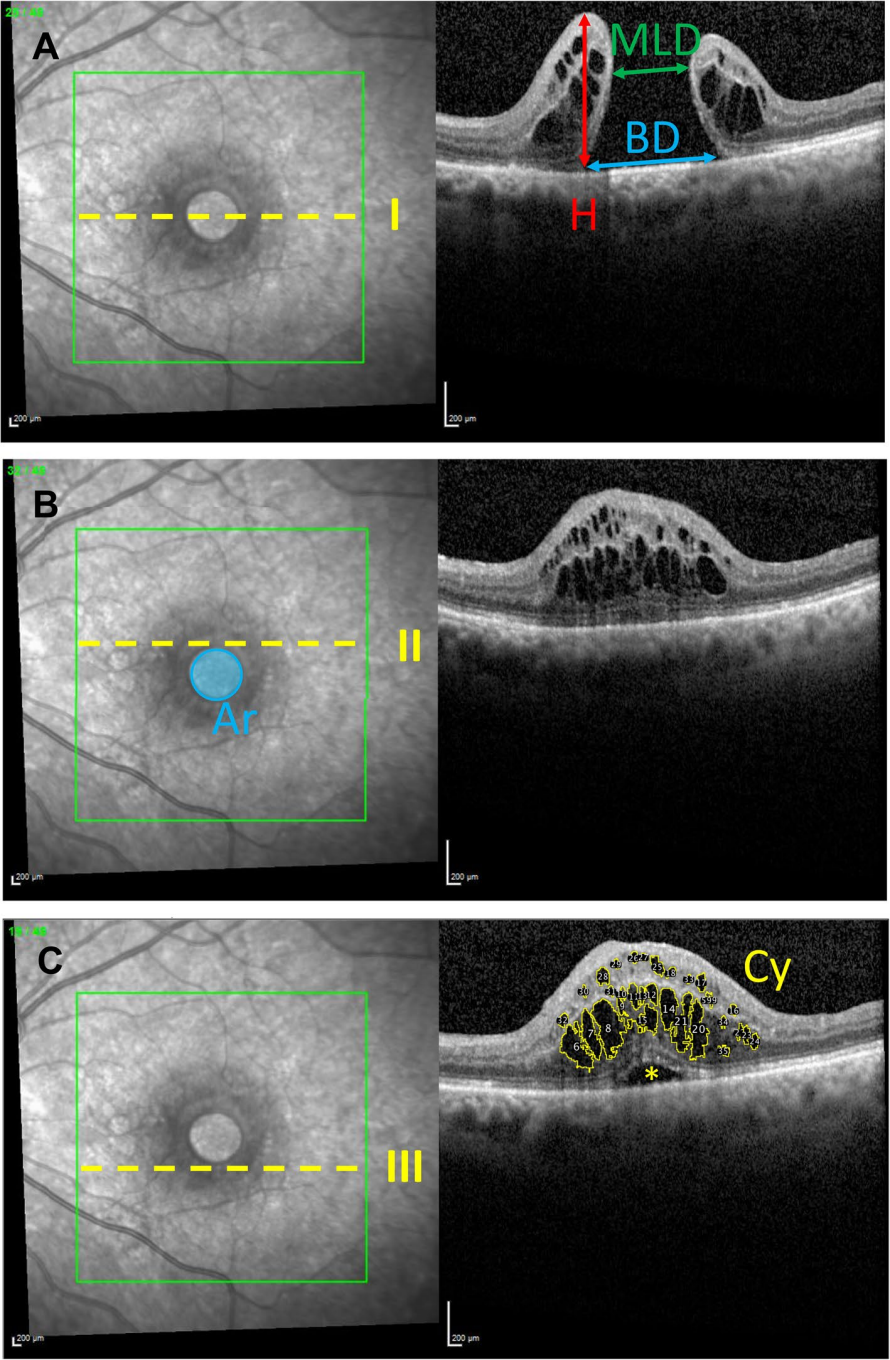
Schematic presentation of the presurgical OCT-based measurements. **A** In the MH central cross-sectional image, the minimum linear diameter (MLD; green arrow), base diameter (BD; blue arrow) and height (H; red arrow) were measured. **B** In the *fundus control image* of the OCT image, the MH area (Ar; blue area) was manually marked and calculated in mm^2^. **C** The areas of the intraretinal pseudocysts (Cy, yellow areas) were measured in three cross-sectional OCT images (I, II and II). Subfoveal fluid (yellow asterisk) was excluded from the calculation of the area (**C**). The yellow broken lines represent the location of the scans (I: central through MH, II and III superior and inferior tangential to MH, respectively)

The following data were collected in the clinical records: age at surgery, sex, and duration from onset of symptoms until surgery. Best corrected visual acuity (VA) was assessed with standard optotypes at a 5 m distance before surgery and at a follow-up examination scheduled for 6 weeks after surgery. Axial length was measured with an IOL Master (Zeiss, Oberkochen, Germany). In the MH central cross-sectional image, taken with optic coherence tomography (OCT; Spectralis-OCT, Heidelberg Engineering, Germany) before surgery, the minimum linear diameter *(MLD)*, the base diameter *(BD)* and the height (*H*) were measured, and the macular hole index (MHI: *max. Height/max. diameter*), diameter hole index (DHI: *min. Diameter/max. diameter*) and tractional hole index (THI: *max. Height/min. diameter*) were calculated as previously described [6, 9, 11–14] (Fig. 1 A). For the minimum linear diameter (MLD), the minimum distance between the inner edges of the MH was measured parallel to the retinal pigment epithelium (RPE). The base diameter (BD) was defined as the diameter at the level of the RPE. The height (H) was measured as the distance from the RPE to the innermost aspect of the MH. In a few cases of atypical MH, e.g., with a distinctly curved or skewed RPE, we plotted an approximated tangent line at the RPE and measured the diameters parallel to this line. In the fundus control image of the OCT image, the MH area (Ar) was manually marked and calculated in mm^2^ (Fig. 1B). The areas of the intraretinal pseudocysts (Cy) were measured in three cross-sectional OCT images (I: central to MH, II and III superior and inferior tangential to MH). The area was manually marked as a region of interest (ROI) in ImageJ (V. 1.52a; National Institutes of Health; Bethesda, Md., USA) and calculated as the sum of all ROIs per patient in pixels. Subfoveal fluid was excluded (Fig. 1C). Furthermore, all OCT images were examined for the presence of epiretinal membrane and vitreomacular traction.

Surgeries were performed by three surgeons with a comparable, very high level of experience. The surgical procedure was almost identical among the three surgeons (total pars plana vitrectomy, membrane peeling after application of indocyanine green (ICG) dye, postvitrectomy gas exchange with perfluoroethane gas) except for minor differences, e.g., only two surgeons regularly stained the vitreous with triamcinolone for better visualization. If indicated, phacoemulsification with intraocular lens implantation was performed before pars plana vitrectomy. After surgery, face-down positioning was recommended for 10 to 14 days as much as possible.

### Statistical evaluation

Statistical analysis was performed using Prism 9.0.0 (GraphPad, La Jolla, California, USA). The normality of the distribution of the data was analyzed with the Shapiro–Wilk test. Data are presented descriptively with the median and interquartile range (presented as the *median (25th percentile; 75th percentile)*). The visual acuity before treatment and at follow-up were compared with the nonparametric Wilcoxon matched pairs signed-rank test. For group comparisons, the Mann–Whitney U test was performed. Fisher’s exact test, the chi-squared test and Spearman’s R were used to investigate correlations. *P* values ≤0.05 were considered statistically significant. Linear and logistic regression analyses were performed on parameters that correlated highly significantly (min. *p* ≤ 0.01) with postoperative VA, and that were highly significantly (min. p ≤ 0.01) different between the *MH closed* and *MH persisting groups*. In the regression models, the macular hole indices were omitted due to multicollinearity/redundancy of the data. In the linear regression analysis of the influence on the postoperative VA, only patients with a closed MH were included so that only parameters that had a direct influence on visual prognosis and not on surgical failure with a subsequently significantly worse VA were evaluated.

## Results

**Fig. 2:**
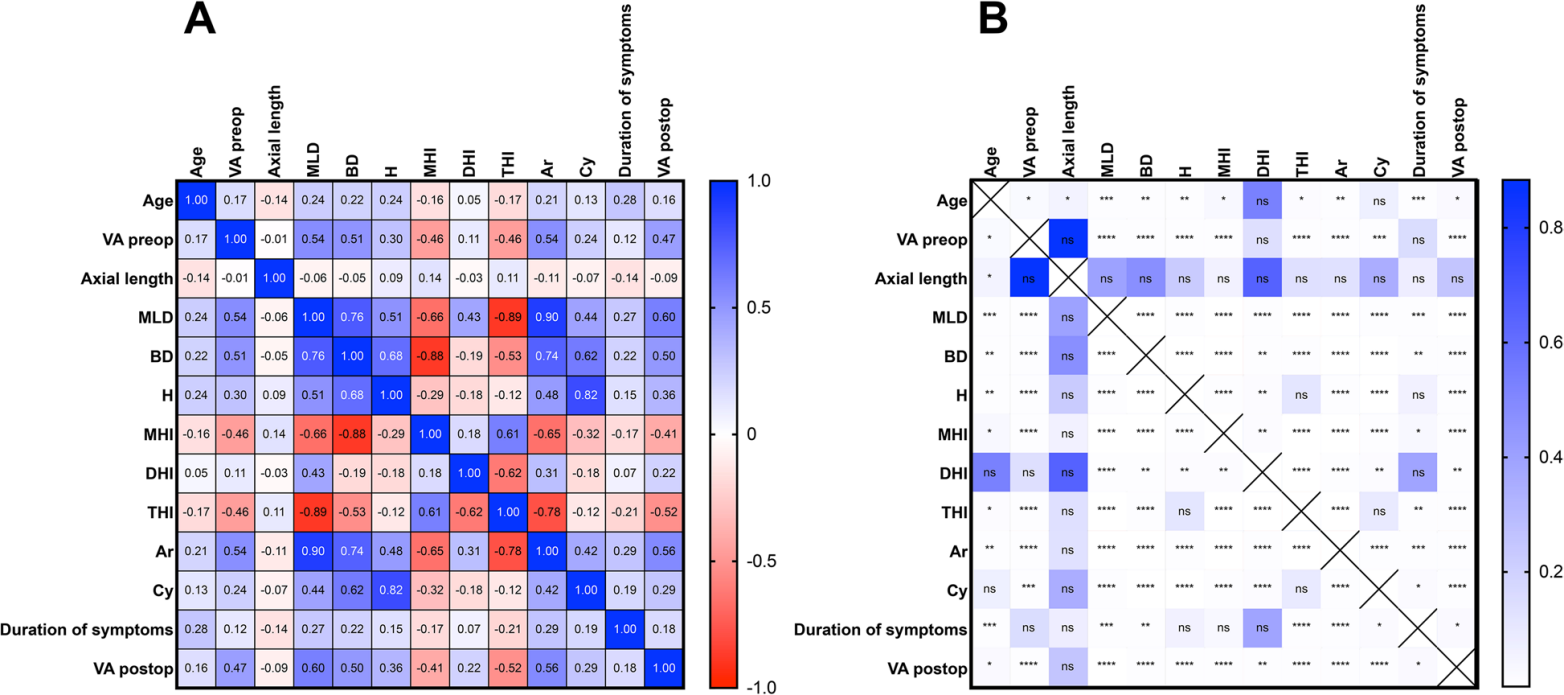
Heatmap and correlation matrix of all analyzed numeric parameters (**A**) and the respective significance levels (**B**). The stars symbolize statistical significance in the gradations; *p* ≤ 0.05 (*), *p* ≤ 0.01 (**), *p* ≤ 0.001 (***) and *p* ≤ 0.0001 (****). This map demonstrates that apart from axial length, there are numerous significant interdependencies and correlations between almost all clinical- and OCT-based parameters. Abbreviations: MLD: minimum linear diameter; BD: base diameter; H: height; MHI: macular hole index; DHI: diameter hole index; THI: tractional hole index; Ar: MH area, Cy: cyst area; VA: visual acuity; ns: not significant

A total of 189 eyes of 178 patients (71.4% female; mean age 67.5 ± 8.2y) who underwent surgery because of MH were included in the analysis. The ﻿female patients were on average younger (female: 66.6 ± 8.0 vs. male: 69.8 ± 8.2; *p* = 0.014). There were no sex-dependent differences in the other preoperative parameters or the closure rate. As the standard follow-up examination was scheduled for 6 weeks after surgery, the median follow-up time was 6 (6; 6) weeks, with few deviations, ranging from 4 to 10 weeks. The overall closure rate was 86.8% (*n* = 164). The mean best corrected visual acuity (VA) was 0.7 ± 0.3 logMAR before surgery, which significantly increased to 0.5 ± 0.3 logMAR postoperatively (*p* < 0.0001). Younger age showed a significant but weak correlation with better pre- and postoperative VA (age vs. VA preop: *p* = 0.0256; *r* = 0.1668; age vs. VA postop: *p* = 0.0256; *r* = 0.1628; see Fig. 2). While initially there was no difference in VA between males and females, postoperative VA was marginally worse in females (final VA female: 0.5 (0.3;0.8) vs. final VA male: 0.4 (0.275;0.6); *p* = 0.044). Each of the three surgeons treated approximately one-third of the cases (surgeons A 38%, B 33% and C 28%). There was no difference in initial and final VA or in surgical success between the three surgeons. The use of triamcinolone and the lens status did not influence the closure rate or outcome of visual acuity.

**Fig. 3:**
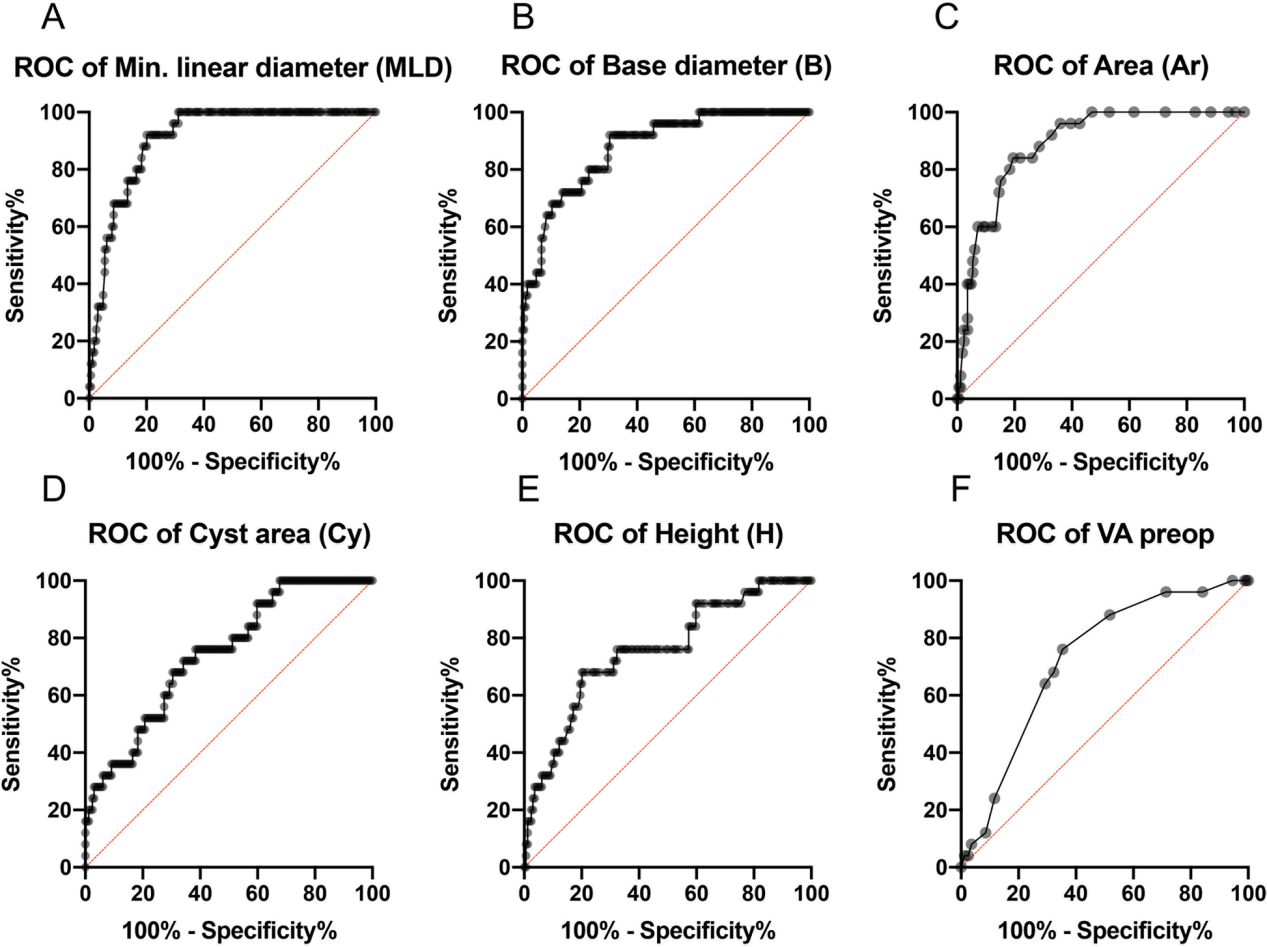
Receiver operating characteristic (ROC) curves for the parameters evaluated in the multiple logistic regression predicting surgical success (MH closed). The ROC curve of the minimum linear diameter indicated that it had the strongest predictive value. (The nearer the ROC curve reaches the upper-left corner of the graph, the larger the area under the ROC curve (AUROC) is. A larger area under the curve represents a higher false-negative rate and a lower false-positive rate and thus a high sensitivity and specificity. Diagonal segments are produced by ties.)

When comparing patients with a closed MH (*MH closed* group) to those with a persisting MH (*MH persisting* group) after surgery, there was no difference in sex distribution (Table 1). However, with respect to age, the two groups differed significantly. Patients with MH closure were on average 67 ((62; 72), range 49–91) years old, while those with a postoperative persisting macular hole were on average 70 ((63; 79) min. 43 max. 88) years old (*p* = 0.033). Additionally, in the *MH closed* group*,* the preoperative VA was significantly better than that in the *MH persisting* group (*MH closed*: 0.7 (0.5; 1,0) min. 0,1, max. 1,5 vs. *MH persisting*: 1.0 (0.8; 1.0) min. 0.4, max. 1.5; *p* = 0.0002). Naturally, the postoperative VA differed significantly between the *MH closed* (0.4 (0.3; 0.6) min. 0, max. 1,4) and *MH* persisting (0.9 (0.8; 1.1) min. 0.5, max. 1.9) groups (*p* < 0.0001). The difference in the duration of symptoms between the *MH closed* and *MH persisting* groups can be interpreted as a trend *(MH closed*: 42 (21; 120) days; min. 1 day, max. 720 days vs. *MH persisting* 105 (21; 270) days; min. 14 days, max. 720 days; *p* = 0.097). The comparison of the morphologic parameters revealed differences between the groups for the minimum linear diameter (MLD) *(MH closed*: 281.5 (198; 371) μm vs. *MH persisting:* 559 (444; 633) μm; *p* < 0.0001), the base diameter (BD) *(MH closed*: 752 (522;958) μm vs. *MH persisting:* 1.219 (1028; 1668) μm; *p* < 0.0001) and the maximal height (H) (*MH closed*: 422.5 (365; 477) vs. *MH persisting:* 506 (462; 594); *p* < 0.0001). Fifty-three MHs (28,0%) had an MLD of 400 μm or greater (22/53 open), and 30 MHs (15,8%) had an MLD of 500 μm or greater (16/30 open). Of the indices based on these measurements, only MHI and THI differed between the two groups (MHI: *MH closed*: 0.61 (0.48; 0.76) vs. *MH persisting:* 0.39 (0.35; 0.49); *p* < 0.0001); THI: *MH closed*: 1.55 (1.20; 2.05) vs. *MH persisting:* 0.90 (0.74; 1.17); *p* < 0.0001), while DHI did not. Additionally, the area of the MH (Ar) and of the intraretinal pseudocysts (Cy) were larger in *MH persisting* (Ar: *MH closed*: 0.08 (0.05; 0.15) mm^2^ vs. *MH persisting:* 0.27 (0.19; 0.36) mm^2^; *p* < 0.0001; Cy: *MH closed*: 6.122.5 (2.667; 10.228) pixel vs. *MH persisting:* 10.851 (8.057; 19.008) pixel; *p* < 0.0001). Vitreomacular traction (VMT) was significantly more frequent in MH closed (*MH closed*: 27.8%, (*n* = 45 of 164 closed MHs) vs. *MH persisting:* 8% (*n* = 8 of 25 persisting MHs); *p* = 0.036). The median axial length was *23.39 (22.87;24.19) mm.* There was no difference in the presence of the epiretinal membrane or axial length between the two groups. Multiple logistic regression analysis showed that the minimum linear diameter was the strongest predictive factor for surgical success (Table 2). Additionally, ﻿receiver operating characteristic (ROC) curve analysis showed the highest area under the curve value for the minimum linear diameter (Fig. 3 and Table 3).

**Table 1:** Overview of the evaluated parameters

	Overall		Macular holes closed		Macular holes open		
		n		n		n	p
	–	189	86.7%	164	13.2%	25	–
**Age (years)**	67 (63; 72) min. 43, max. 91	–	67 (62; 72) min. 49, max. 91	–	70 (63; 79) min. 43, max. 88	–	0.033^#^
**Female**	71.4%	135	70.7%	116	76.0%	19	0.812^§^
**Axial length (mm)**	23.23 (22.52; 23.64) min. 23.44, max. 31.71)	–	23.44 (22.95; 24.37) min. 20.97, max. 31.71)	–	23.23 (22.52; 23.64) min. 20.9, max. 28.12)	–	0.061^#^
**Pseudophakia**	27.5%	52	25.6%	42	40.0%	10	0.153^§^
**Duration of symptoms (days)**	49 (21; 150) min. 1, max. 720	–	42 (21; 120) min. 1, max. 720	–	105 (21; 270) min. 14, max. 720	–	0.097^#^
**VA preop (logMAR)**	0.7 (0.5; 1.0) min. 0.1, max. 1.5	–	0.7 (0.5; 1.0) min. 0.1, max. 1.5	–	1.0 (0.8; 1.0) min. 0.4, max. 1.5	–	0.0002^#^
**MLD (μm)**	299 (209; 414.5) min. 19, max. 1.067	–	281.5 (198; 371) min. 19, max. 914	–	559 (444; 633) min. 342, max. 1.067	–	< 0.0001^#^
**BD (μm)**	793 (537.5; 1046) min. 163, max. 2147	–	752 (522; 958) min. 163, max. 1674	–	1219 (1028; 1668) min. 622, max. 2.147	–	< 0.0001^#^
**H (μm)**	432 (374; 492) min. 203, max. 819	–	422.5 (365; 477) min. 203, max. 819	–	506 (462; 594) min. 336, max. 763	–	< 0.0001^#^
**MHI**	0.56 (0.44; 0.75) min. 0.24, max. 1.72	–	0.61 (0.48; 0.76) min. 0.24, max. 1.72	–	0.39 (0.35; 0.49) min. 0.25, max. 0.67	–	< 0.0001^#^
**DHI**	0.41 (0.31; 0.50) min. 0.03, max. 0.92	–	0.39 (0.30; 0.50) min. 0.03, max. 0.92	–	0.43 (0.37; 0.55) min. 0.21, max. 0.72	–	0.185^#^
**THI**	1.43 (1.10; 1.97) min. 0.47, max. 19.27	–	1.55 (1.20; 2.05) min. 0.47, max. 19.27	–	0.90 (0.74; 1.17) min. 0.58, max. 1.65	–	< 0.0001^#^
**Ar (mm2)**	0.09 (0.06; 0.18) min. 0.01, max. 1.26	–	0.08 (0.05; 0.15) min. 0.01, max. 1.26	–	0.27 (0.19; 0.36) min. 0.09, max. 0.96	–	< 0.0001^#^
**Cy (pixels)**	6897 (2977; 10,643) min. 0, max. 31,383	–	6122.5 (2667; 10,228) min. 0, max. 25,375	–	10,851 (8057; 19,008) min. 3388, max. 31,383	–	< 0.0001^#^
**Vitreomacular traction**	24.8%	47	27.4%	45	8.0%	2	0.036^§^
**Epiretinal membrane**	24.8%	47	23.9%	39	32.0%	8	0.315^§^
**Surgeon A/B/C**	A: 28.0%/B: 38.6%/C: 33.3%	A: 53/B: 73/C:63	83.0%/84.9%/92.1%	A: 44/B: 62/C: 58	A: 17%/B: 15.1%/C: 7.9%	A: 9/B: 11/C: 5	0.301^$^
**Triamcinolon use**	64.00%	121	87.6%	106	12.4%	15	0.66^§^
**VA postop (logMAR)**	0.4 (0.3; 0.7) min. 0, max. 1.9	–	0.4 (0.3; 0.6) min. 0, max. 1.4	–	0.9 (0.8; 1.1) min. 0.5, max. 1.9	–	< 0.0001^#^

Overview of the evaluated parameters in the total cohort as well as a comparison between the parameters in the MH closed vs. MH persisting groups. All data except percentages are presented as the median with IQR (median (Q1; Q3)) and range (min., max.). Statistical tests applied: ^#^ Mann–Whitney U test; ^$^ Chi-square test; ^§^ Fisher’s exact test; Abbreviations: *MLD* minimum linear diameter, *BD* base diameter, *H max*. Height, *THI* tractional hole index, *MHI* macular hole index, *DHI* diameter hole index; *Ar* area of MH, *Cy* areas of pseudocysts, *VA* visual acuity; ns: not significant

**Table 2:** Multiple logistic regression analysis of factors ﻿predicting surgical success (MH closed)

Parameter	Coefficient	95% CI	p	Sign. level
**MLD**	0.006	0.001678 to 0.01249	0.015	*
**BD**	0.002	-4.239e-005 to 0.005160	0.068	ns
**H**	0.001	−0.007413 to 0.008960	0.735	ns
**Ar**	− 0.569	−8.378 to 3.570	0.835	ns
**Cy**	< 0.001	−6.339e-005 to 0.0001567	0.407	ns
**VA preop**	−0.231	−2.465 to 2.030	0.839	ns

In multiple logistic regression analysis, only parameters with highly significant differences (min. p ≤ 0.01) between the *MH closed* and *MH persisting* groups were included, and macular hole indices were omitted due to multicollinearity/redundancy of the data. The aperture diameter was the strongest predictive factor for surgical success. Abbreviations: *CI* confidence interval, *MLD* minimum linear diameter, *BD* base diameter, *H max.* Height, *Ar* area of MH, *Cy* area of pseudocysts, *VA* visual acuity; ns: not significant; *: *p* ≤ 0.05

**Table 3:** ROC curve data and cutoff value

Parameter	AUROC	95% CI	Cutoff value	Youden’s index (J)	Sensitivity (%)	Specifity (%)	p
**MLD**	0.9078	0.8624 to 0.9532	> 392.5	0.72	92	79.9	< 0.0001
**BD**	0.8757	0.8078 to 0.9437	> 861.5	0.61	92	69.5	< 0.0001
**H**	0.7548	0.6506 to 0.8589	> 484.5	0.48	68	79.9	< 0.0001
**Ar**	0.8863	0.8308 to 0.9419	> 0.1650	0.64	84	80.5	< 0.0001
**Cy**	0.7427	0.6458 to 0.8396	> 3337	0.32	100	32.3	< 0.0001
**VA preop**	0.7256	0.6324 to 0.8188	> 0.7500	0.40	76	64.6	0.0003

Table 3 shows the area under the curve (AUROC) and confidence interval (CI) of the receiver operating characteristic (ROC) curves (see Fig. 3) for the prognostic parameters analyzed in the regression models. The cutoff value was derived according to the maximum value of Youden’s index (J). Sensitivity and specificity show the highest possible values at the cutoff value. Abbreviations: *CI* confidence interval, *MLD* minimum linear diameter, *BD* base diameter; *H max.* Height, *Ar* area of MH, *Cy* area of pseudocysts, *VA* visual acuity

Analogous to the surgical outcome, the morphologic parameters also had an influence on postoperative VA. Thus, a lower minimum linear and base diameter, a lower maximal height, a lower MH area, and lower areas of pseudocysts was correlated with a higher postoperative VA (Fig. 2). Among the indices, a lower MHI and THI was correlated with a higher VA, while DHI inversely was correlated with postoperative VA. (Fig. 2).

Additionally, the morphological parameters were correlated with each other, as well as with age, duration of symptoms, and pre- and postoperative VA did for the most part (Fig. 2). In contrast, there was no correlation of the axial length with morphological or other quantitative parameters (see Fig. 2). MHs with VMT showed better final VA than those without VMT (final VA with VMT: 0.4 (0.3; 0.5) vs. final VA without VMT: 0.5 (0.3; 0.8); *p* = 0.003). Linear regression analysis showed that initial VA and the minimum linear diameter were correlated with final VA (Table 4).

**Table 4:** Multiple linear regression analysis of factors ﻿predicting postoperative VA

Parameter	Coefficient	95% CI	p	Sign. level
**MLD**	< 0.001	0.0003087 to 0.001009	< 0.001	***
**BD**	< 0.001	−0.0001642 to 0.0002001	0.761	ns
**H**	< 0.001	−0.0004810 to 0.0006534	0.750	ns
**Ar**	0.234	−0.2975 to 0.4503	0.196	ns
**Cy**	< 0.001	−9.104e-006 to 8.774e-006	0.739	ns
**VA preop**	0.190	0.1308 to 0.4241	0.009	**

In multiple linear regression analysis, only parameters with highly significant correlations (min. *p* ≤ 0.01) with postoperative visual acuity and only patients with a closed MH (*n* = 164) were included. Macular hole indices were omitted due to multicollinearity/redundancy of the data. The aperture diameter and preoperative visual acuity were the strongest predictive factors for postoperative visual acuity. Abbreviations: *CI* confidence interval, *MLD* minimum linear diameter, *BD* base diameter; *H max.* Height, *Ar* area of MH, *Cy* area of pseudocysts, *VA* visual acuity, *ns* not significant; **: *p* ≤ 0.01; ***: *p* ≤ 0.001

## Discussion

### Age/sex

The higher proportion of females in our cohort as well as the mean age is consistent with previous reports [2, 6, 9, 11, 15–18]. In our cohort, younger age was correlated with better visual and surgical outcomes, which is in line with previous studies [16, 17, 19]. In contrast, in the works of Yuksel et al., Zou et al. and Steel et al., age was not a predictive factor for outcome [2, 9, 20]. This difference might be explained by differences in the study settings and the development of visual acuity after surgery over time. As Tirelli et al. showed, the increase in visual acuity takes longer in older patients [21]. Thus, the final visual acuity depends on the time period between surgery and the last follow-up. In our study, this was a median of 6 weeks; in the study by Gupta et al., it was 3 months [17]. Yuksel et al., Zou et al. and Steel et al., on the other hand, had a follow-up period of up to 12 months [2, 9, 20].

### Duration of symptoms

﻿The influence of the duration of preoperative symptoms was analyzed by several other study groups, and they reported results similar to ours [2, 3, 22]. The duration of symptoms thus seems to be a suitable and simple survey parameter, although it might be biased due to its subjective perception.

We did not find a significant correlation between the duration of symptoms and the outcome. Nevertheless, we interpret the differences regarding this parameter between open and closed MH as a trend and thus might show statistical significance in a larger cohort. Fallico et al. proposed that small macular holes with a short duration of symptoms may be treated urgently, as there is a relation between better visual outcome and time from symptom onset to surgery for small macular holes [3].

### Visual acuity

Regression analysis revealed that better preoperative VA was a strong predicator for better VA at the postoperative follow-up. Additionally, in the *MH closed* group, preoperative VA was significantly better than that in the MH persisting group. This is in accordance with previous studies with longer follow-up [2, 3, 7, 17, 22, 23]. Nevertheless, it has been shown that VA increases up to 2 years postoperatively [23, 24]. Furthermore, as already noted above, the duration of postsurgical recovery also seems to be influenced by the patient’s age. Tirelli et al. investigated postoperative visual acuity after 0, 30 and 90 days [21]. They found that younger patients already reached the best possible VA after 30 days, which remained stable thereafter. Older patients, however, required more time to recover and reached the best outcome after 90 days [21]. The follow-up period in our study was limited because the surgeries were performed in a referral hospital, and after one follow-up examination, scheduled at 6 weeks after surgery, the patient was attended by the referring ophthalmologist. In clinical practice, patients with successful anatomical and visual outcomes are unlikely to be followed up long-term. This issue has previously been addressed by Fallico et al., who included only patients who were followed up for at least 1 year [3]. We fully concede that the short follow-up period of this study is one of the main limitations regarding postoperative visual outcome.

### Minimum linear diameter

Although several of the evaluated parameters were correlated with the surgical and visual outcomes in our study, the minimum linear diameter (MLD) was the only parameter showing predictive properties in both the linear and logistic regression analyses. Although the significance of the MLD might be somewhat limited due to poor intra- and interindividual repeatability in manual OCT diameter measurements, as recently shown by Antonopoulou, the correlation of preoperative macular hole diameter and outcome was documented in several earlier studies [2, 3, 9, 16, 22, 25–27]. In our study, the MLD was a stronger predicator for postoperative VA than the basal diameter or the manually marked MH area (Fig. 2). In contrast to Steel et al., we found—as did all other comparable studies before—no significant difference in preoperative hole size between females and males [2]. Furthermore, while our cutoff value for an insufficient surgical outcome (> 392.5 μm) corresponds closely to the cutoff values for large macular holes by the International Vitreomacular Traction Study (IVTS) group, Steel et al. proposed a minimum linear diameter of ~ 500 μm as the threshold where the success rate starts to decline [2, 14]. The receiver operating characteristic curve for surgical failure and MLD in their study reached an area under the curve (AUROC) of 77.9%; in our study, it reached 90.1%. Ch’ng et al. proposed even higher threshold values for large MHs. They reported a cutoff value of 630 μm, yielding a Youden index (J) of 0.46 [28]. The Youden Index is a frequently used summary measure of the ROC curve. It both measures the effectiveness of a diagnostic marker (here: MLD) and enables the selection of an optimal cutoff point for this marker to determine the outcome (here: MH closed vs. MH persisting). It ranges between 0 and 1. A value of 0 indicates that it has no predictive value, and a value of 1 represents the perfect test or biomarker. A Youden index (J) of 0.46, as reported by Ch’ng et al., corresponds to a relatively low to moderate level of effectiveness/separation. In contrast, the Youden index for our cutoff of 392 μm was 0.72, corresponding to a very good level of effectiveness/separation (see Table 3).

### VMT

It is generally known that increased vitreomacular adhesion can lead to VMT and subsequently to a macular hole due to anterior and posterior traction forces [29–33]. In contrast to Philippakis et al. [34], we found a marginally significantly higher closure rate (*p* = 0.036) and significantly better visual acuity (*p* = 0.003) in patients with VMT than in patients without VMT. However, Philippakis et al. (*n* = 77 eyes) assumed that a larger study population would have resulted in significant differences [34]. One explanation for the better morphological and functional outcome in our study could be that patients with VMT presented with beneficial factors such as lower overall age and smaller hole size. Thus, it is not useful as a prognostic indicator.

### Perifoveal pseudocysts

﻿The areas of parafoveal intraretinal pseudocysts were correlated in our study with a low closure rate and with low postoperative visual acuity. In the study by Ruiz-Moreno et al., the mean pre- and postoperative VA was lower in patients with cystic retinal changes than in those without [6]. Yuksel et al. also found a correlation of pseudocysts and persistent macular holes but no correlation with postoperative visual acuity [9]. In contrast, Brockmann et al. found that the presence of parafoveal pseudocysts was associated with a higher closure rate. However, in their work, the presence of perifoveal pseudocysts was only assessed qualitatively [10]. In a similar approach by Chhablani et al., the presence of cystic edges was associated with anatomical success and a better final VA [12]. ﻿Liang et al. suggested that a cystic configuration might contribute to spontaneous closure of the macular hole [1]. Venkatesh et al. calculated the macular hole cystoid space area index (MCSAI; MCSAI = ﻿macular hole cystoid space area/total MH area) and ﻿showed its possible predictive value.

### Lens status

Consistent with our findings, several previous studies did not find a significant impact of lens status and macular hole surgery combined with cataract surgery on anatomical or visual outcomes [2, 9, 18, 19]. Essex et al., however, found that combined phacovitrectomy was associated with better VA postoperatively (vs. vitrectomy surgery alone), a difference that vanished when eyes went on to have subsequent cataract surgery [23]. Due to the relatively short follow-up in our study, phakic patients had not developed visually significant cataracts, as in the work by Steel et al. [2].

### Indices

In our study, MHI, DHI and THI were correlated with postoperative visual acuity (VA), although DHI showed a rather weak correlation. In the literature, the results regarding the correlation of the indices and final VA are mostly comparable. In several studies, a correlation between MHI and postoperative VA was shown [6, 8, 9, 11, 12]. Additionally, a correlation with the tractional index THI has been described [6, 8, 12]. Nevertheless, there are some controversial results. While Chablani et al. also confirmed the correlation of THI with VA, in their study, there was no correlation between final VA and MHI and DHI. Ruiz-Moreno et al. found that MHI and THI, but not DHI, were correlated significantly with postoperative VA [6].

Regarding differences in the indices in the surgical outcome group, Wakely et al. found an association between anatomical success and MHI but not THI [8]. Venkatesh et al. showed that MHI and THI were capable of predicting anatomical success [35]. Chhablani et al. found a significant correlation of all three indices (THI, MHI and DHI) with anatomical success [12].

### Limitations

As mentioned above, the main limitation of our study is the short follow-up time. Additionally, the size of our cohort might be considered a limiting factor. While there are some studies with larger cohorts in general, there are only a few with larger cohorts with such an extensive analysis of parameters. To our knowledge, our study is the largest cohort for some of the analyzed parameters, such as intraretinal pseudocysts. Nevertheless, some parameters regarding the macular hole anatomy, such as the hole angle, volume and base area, were not evaluated [36–38]. Additionally, other very specific details of retinal changes, e.g., in the ﻿﻿ellipsoid zone, photoreceptor structure, ganglion-cell thickness and choroidal thickness and perfusion, have not been studied [39–42]. Furthermore, the inclusion of 11 patients with MH in both eyes could possibly result in a statistical bias caused by correlations associated with binocularity. To rule out such a bias, all results were recalculated without those patients. This did not change the significance levels or the results themselves. For this reason, binocular patients were not excluded.

## Conclusion

The aim of this retrospective study was to analyze factors influencing the outcome of surgically treated MH and to identify the factor with the strongest predictive power. The minimum linear diameter was the only parameter showing predictive properties in the regression analyses regarding both surgical and functional outcome. Thus, in our opinion, the results of this study show that there is no need for complicated measurements of the macular hole area or cyst areas or for the calculation of any indices. The minimum linear diameter serves as an easily assessed parameter with the best predictive properties. This result is of great importance for clinical practice, as it simplifies the postsurgical prognosis.

## Data Availability

The datasets used and/or analyzed during the current study are available from the corresponding author on reasonable request.
